# Yeast recombination-based cloning as an efficient way of constructing vectors for *Zymoseptoria tritici*

**DOI:** 10.1016/j.fgb.2015.03.017

**Published:** 2015-06

**Authors:** S. Kilaru, G. Steinberg

**Affiliations:** School of Biosciences, University of Exeter, Exeter EX4 4QD, UK

**Keywords:** YRBC, yeast recombination-based cloning, *sdi1*, succinate dehydrogenase, *hph*, hygromycin phosphotransferase, *nptII*, neomycin phosphotransferase, *bar*, bialaphos resistant gene, GFP, green-fluorescent protein, Zt, *Zymoseptoria tritici*, RB and LB, right and left border, bp, base pairs, Selectable markers, Hygromycin, Geneticin, Carboxin and BASTA, *Septoria tritici* blotch, *Mycosphaerella graminicola*

## Abstract

Many pathogenic fungi are genetically tractable. Analysis of their cellular organization and invasion mechanisms underpinning virulence determinants profits from exploiting such molecular tools as fluorescent fusion proteins or conditional mutant protein alleles. Generation of these tools requires efficient cloning methods, as vector construction is often a rate-limiting step. Here, we introduce an efficient yeast recombination-based cloning (YRBC) method to construct vectors for the fungus *Zymoseptoria tritici*. This method is of low cost and avoids dependency on the availability of restriction enzyme sites in the DNA sequence, as needed in more conventional restriction/ligation-based cloning procedures. Furthermore, YRBC avoids modification of the DNA of interest, indeed this potential risk limits the use of site-specific recombination systems, such as Gateway cloning. Instead, in YRBC, multiple DNA fragments, with 30 bp overlap sequences, are transformed into *Saccharomyces cerevisiae*, whereupon homologous recombination generates the vector in a single step. Here, we provide a detailed experimental protocol and four vectors, each encoding a different dominant selectable marker cassette, that enable YRBC of constructs to be used in the wheat pathogen *Z. tritici*.

## Highlights

• Yeast recombination-based cloning (YRBC) is a reliable and inexpensive way of generating plasmids.
• We provide 4 vectors for YRBC that a cover different resistance genes.
• Using this technique promises rapid generation of molecular tools to study *Z. tritici*.

## 1 Introduction

*Zymoseptoria tritici* is a dimorphic ascomycete fungus, which ranges amongst the most wheat pathogens in Europe (Dean et al., 2012; Gurr and Fones, 2015). Developing new strategies to control this pathogen requires in-depth knowledge of its invasion strategy and insight into crucial cellular processes required for growth and proliferation. Such progress is strongly dependent on development of molecular tools and techniques. Previous work provided transformation protocols, vectors with different dominant selectable markers, conditional promoter analysis, GFP reporter system, virulence assays and high-throughput automated image analysis for *Z. tritici* (Bowler et al., 2010; Kema et al., 2000; Perez-Nadales et al., 2014; Rohel et al., 2001; Rudd et al., 2008; Skinner et al., 1998; Stewart and McDonald, 2014; Zwiers and De Waard, 2001). However, to further accelerate progress and extend the repertoire of molecular tools, efficient cloning methods are needed.

The majority of vectors for manipulation of *Z. tritici* have been generated using conventional cloning methods, including the use of restriction enzymes and *in vitro* ligation protocols (Adachi et al., 2002; Choi and Goodwin, 2011; Marshall et al., 2011; Motteram et al., 2009, 2011; Roohparvar et al., 2007; Zwiers and De Waard, 2001; Zwiers et al., 2007). However, these procedures carry numerous limitations. Firstly, they depend on the availability of unique and compatible restriction sites in the vector and the DNA fragment(s) to be cloned. Indeed, searching for the availability of such restriction sites or introducing new restriction sites in the DNA is time and labour-intensive (Benoit et al., 2006). Furthermore, the various manipulations could modify the primary sequence of the encoded gene product (Andersen, 2011), with the downstream risk of affecting the function of the gene products (Kilaru et al., 2009).

Recently, Gateway recombination technology was used to generate vectors for *Z. tritici* (Bowler et al., 2010; Mirzadi Gohari et al., 2014; Scalliet et al., 2012). The Gateway cloning method is based on the site-specific recombination properties of the bacteriophage lambda and provides a highly efficient way to clone DNA fragments of interest (Hartley et al., 2000; Landy, 1989). Whilst this is a powerful method for molecular cloning, the Gateway technology introduces 25 bp long “attachment sites” that results in an introduction of 8–11 additional amino acids. Such modification of the primary sequence, couple with the relatively high costs of the Gateway site-specific recombination kits, limit use of this cloning method (Engler et al., 2008).

An alternative cloning approach makes use of the ability of *Saccharomyces cerevisiae* to recombine DNA fragments *in vivo* by homologous recombination (Ma et al., 1987; Raymond et al., 1999). Here, DNA fragments, with overlapping sequences, are transformed into *S. cerevisiae* for *in vivo* recombination (Ma et al., 1987). Such overhangs can be as short as 30 bp (Kilaru et al., 2006; Oldenburg et al., 1997; Schuster et al., 2011a) and are added using commercially synthesized primers. This method circumvents both the need for restriction enzymes and expensive commercial kits. Most importantly, yeast recombination-based cloning (YRBC) avoids changes in the primary DNA sequence. Instead, this method allows precise cloning of multiple overlapping DNA fragments in a single step, thereby rapidly generating complex vectors (Andersen, 2011; Shanks et al., 2006). This powerful cloning method, YRBC has been used to construct viral and bacterial vectors (Shanks et al., 2006; Youssef et al., 2011), and indeed, to assemble the entire genome of the prokaryote *Mycoplasma genitalium* d from 25 overlapping DNA fragments (Gibson et al., 2008). In fungi, YRBC has been used in *Coprinopsis cinerea* (Kilaru et al., 2006), and subsequently, to investigate the corn pathogen *Ustilago maydis* (Schuster et al., 2011a) and the rice blast fungus *Magnaporthe oryzae* (Dagdas et al., 2012; Lu et al., 2014).

Here, we introduce the detailed protocol to construct vectors using YRBC. We also provide four vectors, carrying different dominant selectable marker cassettes, suitable for yeast recombination-based construction of vectors for use in *Z. tritici*.

## 2 Materials and methods

### 2.1 Fungal growth conditions and genomic DNA isolation

*Z. tritici* was grown in YG broth (yeast extract, 10 g/l; glucose, 30 g/l) for 3 days at 18 °C with 200 rpm. Three ml of cells were harvested by centrifugation at 13,000 rpm for 2 min and followed by addition of 400 μl of lysis buffer (2% Triton X, 1% SDS, 100 mM NaCl, 10 mM Tris HCl pH-8.0, 1 mM EDTA), 500 μl phenol: chloroform (1:1) and a small scoop of acid washed glass beads (425–600 μm; Sigma–Aldrich, Gillingham, UK). The tubes were mixed for 10 min by using IKA Vibrax shaker (IKA, Staufen, Germany) and centrifuged for 10 min at 13,000 rpm. The supernatant was transferred to a fresh Eppendorf tube containing 1 ml of 100% ethanol. The tubes were centrifuged for 10 min at 13,000 rpm and the DNA was washed with 500 μl of 70% ethanol. The residual ethanol was removed by incubating the tubes at 55 °C for 5 min and DNA was suspended in 50 μl water/RNaseA solution. For PCR applications, the genomic DNA was diluted with water by 200 times.

### 2.2 Construction of vectors pCGEN-YR, pCHYG-YR and pCBAR-YR using conventional ligation method

The vectors pCGEN-YR, pCHYG-YR and pCBAR-YR were constructed using conventional restriction digestion and ligation cloning method. The yeast recombination cassette consists of URA3 and 2μ *ori* from plasmid pNEB-hyg-yeast (Schuster et al., 2012) was cloned into the vectors pCGEN (Motteram et al., 2011), pCHYG (Motteram et al., 2009) and pCAMB-BAR (Kramer et al., 2009) resulting in vectors pCGEN-YR, pCHYG-YR and pCBAR-YR respectively. For construction of vector pCGEN-YR, a 8257 bp of vector pCGEN (*Sac*II and *Psi*I fragment) was ligated with 2820 bp fragment of vector pNEB-hyg-yeast (*Sac*II and *Ssp*I fragment). For construction of vector pCHYG-YR, a 8117 bp of vector pCHYG (*Sac*II and *Psi*I fragment) was ligated with 2820 bp of fragment of vector pNEB-hyg-yeast (*Sac*II and *Ssp*I fragment). For construction of vector pCBAR-YR, a 7616 bp of vector pCAMB-BAR (*Bcl*I and *Psi*I fragment) was ligated with 2847 bp of fragment of vector pNEB-hyg-yeast (*Bcl*I and *Dra*I fragment). Primers SK-41 and SK-Sep-137 (Table 1) were used to identify the positive clones and the expected band sizes are 2728 bp, 2588 bp and 2090 bp for vectors pCGEN-YR, pCHYG-YR and pCBAR-YR respectively.

### 2.3 Construction of vector pCCBX-YR using yeast recombination-based cloning

Plasmid pCCBX-YR was constructed using *in vivo* recombination in the yeast *S. cerevisiae* DS94 (MATα, *ura3-52*, *trp1-1*, *leu2-3*, *his3-111*, and *lys2-801* (Tang et al., 1996) following published procedures (Raymond et al., 1999). For the recombination events, the fragments were amplified with 30 bp homologous sequences to the upstream and downstream of the fragments to be cloned. The detailed steps involved in the construction of this vector are described below.

### 2.4 Primer designing and PCR amplification of DNA fragments

Primer design is vital step in constructing the vectors using YRBC. The 30 bp overlapping sequences to the next DNA fragment needs to be incorporated in the 5′ end of the 20–25 bp primer sequence, which makes the total primer length to 50–55 bp. Likewise, primers SK-Sep-11, SK-Sep-12, SK-Sep-282 and SK-Sep-283 (Table 1) were synthesized and then the desired DNA fragments were amplified either from *Z. tritici* IPO323 (Goodwin et al., 2011; Kema and van Silfhout, 1997) genomic DNA using Phusion high-fidelity DNA polymerase (Thermo Scientific, Leicestershire, UK). PCR was performed by using 1 μl of template DNA with final concentration of 200 μM each dNTPS, 0.5 μM of each oligos, 1x HF buffer, 0.02 U/μl of Phusion DNA polymerase in a total volume of 50 μl. Cycling parameters were 94 °C for 2 min, then 35 cycles of 94 °C for 10 s, 60 °C for 20 s and 72 °C for 2 min (30 s for 1 kb of DNA), followed by a single 10 min extension at 72 °C. The DNA bands of interest were excised and purified from the gel as described below. In parallel, the plasmid to be cloned was digested with suitable restriction enzymes and the DNA fragment of interest excised from the agarose gel.

### 2.5 Purification of DNA fragments

DNA fragments of interest were purified using silica glass suspension as described previously (Boyle and Lew, 1995). In brief, the gel slice was melted at 55 °C for 5 min with 3 volumes of 6 M sodium iodide, followed by further incubation for 5 min at 55 °C with 20 μl silica glass suspension (100 mg/ml stock solution, Sigma–Aldrich, Gillingham, UK). Then, the reaction mixture was centrifuged at 13,000 rpm for 30 s and the supernatant was discarded. The pellet was washed with DNA wash buffer (50 mM NaCl, 10 mM Tris HCl pH-7.5, 2.5 mM EDTA and 50% ethanol (v/v)) for 3 times. Finally, the DNA was eluted from the glass beads by addition of 10 μl water and incubation at 55 °C for 10 min.

### 2.6 Preparation of yeast competent cells and transformation

Transformation of DNA fragments into *S. cerevisiae* DS94 was performed as described previously (Gietz and Schiestl, 2007; Raymond et al., 1999). In brief, the *S. cerevisiae* DS94 cells were grown in 3 ml YPD media (yeast extract, 10 g/l; peptone, 20 g/l; glucose, 20 g/l; agar, 20 g/l) at 28 °C for overnight with 200 rpm. Then, the overnight culture was transferred to 50 ml YPD and grown for 5 h at 28 °C with 200 rpm. The cells were harvested by centrifugation at 2200 rpm for 5 min and cells were washed with 5 ml sterile water. The cells were suspended in 300 μl water and kept at room temperature for further use.

4 μl of each purified DNA fragments of 9,766 bp fragment of pCGEN-YR obtained as *Bam*HI and *Zra*I, 1929 bp PCR product obtained with primers SK-Sep-282 and SK-Sep-11 (Table 1) and 303 bp PCR product obtained with primers SK-Sep-12 and SK-Sep-283 (Table 1) were added to a fresh Eppendorf tube followed by 50 μl salmon sperm DNA (2 μg/μl stock; Sigma–Aldrich, Gillingham, UK), 50 μl *S. cerevisiae* cells, 32 μl 1 M lithium acetate and 240 μl 50% PEG 4000. The components were mixed by gently inverting the tubes for few times and incubated at 28 °C for 30 min. Heat shock was performed at 45 °C for 15 min and tubes were centrifuged at 2000 rpm for 2 min. The supernatant was gently removed and the pellet was suspended in 150 μl water. Finally, the cell suspension was plated on to yeast synthetic drop-out media which lacks uracil (yeast nitrogen base without amino acids and ammonium sulphate, 1.7 g/l; ammonium sulphate, 5 g/l; casein hydrolysate, 5 g/l; tryptophan, 20 mg/l; agar, 20 g/l) and incubated at 28 °C for 2 days.

### 2.7 Colony PCR on yeast colonies and plasmid DNA isolation from yeast cells

Colony PCR was performed on yeast cells by using DreamTaq DNA polymerase (Thermo Scientific, Leicestershire, UK) in 20 μl total volume. Cycling parameters were 94 °C for 5 min, then 35 cycles of 94 °C for 2 min, 60 °C for 30 s and 72 °C for 1 min (1 min for 1 kb of DNA), followed by a single 10-min extension at 72 °C. Primers SK-Sep-282 and SK-Sep-283 (Table 1) were used to identify the positive clones and the expected band sizes are of 2296 bp. Plasmid DNA was isolated from the positive yeast colonies as described previously with slight modification (Hoffman and Winston, 1987). In brief, the recombinant *S. cerevisiae* cells were grown in 15 ml yeast synthetic drop-out media at 28 °C for overnight and harvested by centrifugation at 3000 rpm for 5 min. Then 200 μl yeast-lysis buffer (2% Triton X-100, 1% SDS, 100 mM NaCl, 10 mM Tris pH-8.0 and10 mM EDTA), 200 μl phenol:chloroform: isoamylalcohol (25:24:1 v/v) and 0.3 g acid washed glass beads (425–600 μm diameter, Sigma–Aldrich, Gillingham, UK) were added and the tubes were vortexed for 5 min using IKA Vibrax shaker (IKA, Staufen, Germany). 200 μl TE buffer (10 mM Tris HCl; 1 mM EDTA, pH-8.0) was added and centrifuged for 5 min at 13,000 rpm. The upper aqueous layer was carefully transferred to a fresh Eppendorf tube and 50 μl 3 M sodium acetate pH-5.5 and 1 ml ethanol was added. The tubes were kept at −20 °C for 15 min and centrifuged at 13,000 rpm for 20 min. The cell pellet was suspended in 400 μl TE and RNaseA (Sigma–Aldrich, Gillingham, UK) and incubated at 37 °C for 15 min. DNA was precipitated by addition of 10 μl 4 M ammonium acetate and 1 ml 100% ethanol. The tubes were centrifuged for 5 min at 13,000 rpm and DNA was washed with 70% ethanol. The residual ethanol was removed by incubating the tubes at 37 °C for 10 min and the DNA was suspended in 20 μl water.

### 2.8 *E. coli* transformation and confirmation by restriction analysis

10 μl of DNA isolated from the *S. cerevisiae* was transformed into *E. coli* DH5α by using “homemade” competent cells. In order to prepare the competent cells, a single *E. coli* colony was grown in 20 ml DYT media for overnight at 37 °C with 200 rpm. 100 μl overnight culture was added to 100 ml fresh DYT with 10 mM MgCl_2_ and incubated at 18 °C with 100 rpm until the optical density reaches to 0.25 (for 48 h). Then, the cells were chilled in ice water for 10 min and centrifuged at 4 °C for 10 min at 5000 rpm. The supernatant was discarded and the pellet was suspended in 60 ml ice cold TB (transformation buffer; 250 mM KCl, 15 mM CaCl_2_, 10 mM PIPES, 55 mM MnCl_2_). The cell suspension was centrifuged at 4 °C for 10 min at 5000 rpm and the cell pellet was suspended in 16 ml TB. Finally 1.2 ml DMSO was added and 50 μl aliquots were frozen in liquid nitrogen and the competent cells were stored at −80 °C. Finally, the plasmid DNA was isolated from the *E. coli* colonies and further confirmed by restriction analysis.

## 3 Technical details and discussion

### 3.1 Steps involved in the construction of vectors using yeast recombination-based cloning

**Fig. 1:**
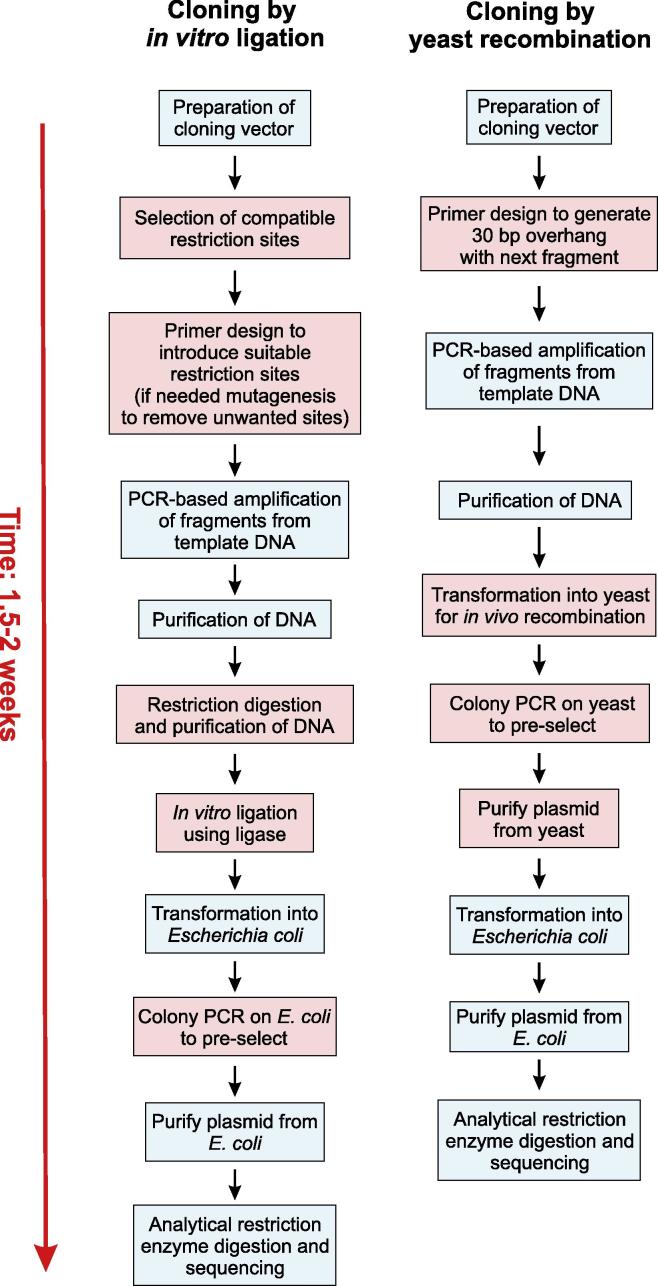
Flow chart depicting experimental cloning steps. YRBC involves fewer steps than standard *in vitro* ligation methods, but requires about the same time input to obtain the final plasmid.

Here, we provide a detailed “step-by-step” description of YRBC and compare the procedure to the more conventional restriction/ligation-based method (Fig. 1). Both methods require digestion of the vector with suitable restriction enzymes. Conventional restriction/ligation-based methods rely on the availability of suitable restriction enzymes, which should generate the compatible ends in both the vector and DNA fragment(s) of interest. By contrast, YRBC requires linearization of the vector, but the enzyme can be chosen freely and independently of the DNA fragment to be cloned (henceforth named “insert”). However, for both methods, the digested vector needs to be purified.

The next step is to design the primers, which may require use of both methods (Fig. 1), depending on the availability of suitable restriction sites in both the vector and the insert. With conventional cloning, such sites are often not present and need to be introduced by polymerase chain reaction (PCR), or existing restriction sites need to be removed by site-directed mutagenesis (Benoit et al., 2006; van den Ent and Lowe, 2006). This results in modifications of the nucleotide sequence. By contrast, YRBC relies on 30 bp homologous sequences, which are added by extended primers to either end of the insert(s). These 30 bp overlapping sequences navigate the recombination event and, therefore, assemble precisely the overlapping DNA fragments, without altering the primary sequence (Oldenburg et al., 1997).

Further steps involve amplification by PCR and the purification of inserts (Fig. 1). In the conventional ligation method, the prepared inserts and the linearized vector are ligated *in vitro*, using DNA ligase. In the YRBC method, the linearized vector and the DNA fragments are transformed into *S. cerevisiae* (Fig. 1), which assembles the vector by *in vivo* recombination. *S. cerevisiae* utilizes its own recombination machinery for this process, circumventing the need for DNA ligase or other commercial cloning kits (Oldenburg et al., 1997). Pre-selection of positive yeast clones is achieved by direct PCR amplification of yeast colonies, followed by purification of plasmid DNA and transformation *E. coli* for *in vivo* amplification. This protocol increases the amount and quality of the DNA (Singh and Weil, 2002).

Finally, both methods require identification of positive *E. coli* transformants and purification of the plasmid for further use.

### 3.2 Comparison of conventional and yeast-based cloning: an example

**Fig. 2:**
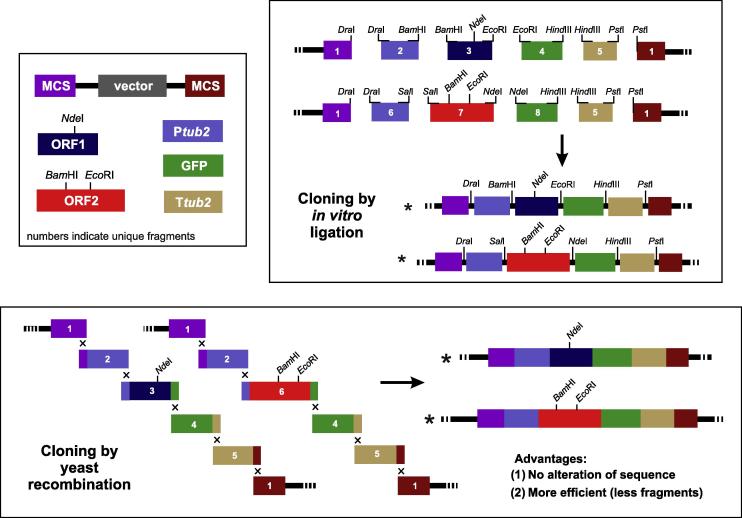
Example cloning strategy showing generation of two GFP-fusion constructs by *in vitro* ligation and YRBC. Due to internal restriction sites, in *vitro ligation* requires introduction of unique restriction sites at the 3′ and 5′ end of each the 8 fragments. Alternatively, unique internal sites can be used (not shown). Both approaches alter the primary sequence (see both final constructs, indicated by asterisks in cloning by *in vitro* ligation). Only a few DNA fragments can be used for cloning both genes (here the cloning vector, fragment 1, and the *tub2* terminator, fragment 5). The yeast recombination method does not involve restriction site generation. Instead, complementary sequence ends of 30 bps are generated that enable homologous recombination in *S. cerevisiae*. The primary sequence is not altered (see final constructs, two asterisks; cloning by yeast recombination), and several fragments can be used for cloning both genes (fragments 1, 2, 4 and 5). Vector = cloning plasmid backbone; MCS = multiple cloning site; P*tub2 *= promoter of the *Z. tritici* alpha tubulin gene *tub2* ; T*tub2 *= terminator of the *Z. tritici* alpha tubulin gene *tub2*; GFP = green fluorescent protein; ORF1, 2 = open reading frames of interest.

The advantages of YRBC over restriction/ligation-based cloning can be best illustrated using a pictorial representation of the construction of two GFP-fusion constructs (Fig. 2). This cloning procedure introduces 4 different inserts (the promoter for alpha-tubulin, P*tub2*; a gene for green-fluorescent protein, *egfp*; an open reading frame for protein 1, *ORF1*; the alpha-tubulin terminator, T*tub2*), cloned into a linearized vector (Fig. 2, multiple cloning sites are indicated by “MCS”), digested, in our example, with *Dra*I and *Pst*I. Following conventional cloning methods, unique restriction sites need to be added to all fragments (see Fig. 2 for example enzymes). Most often, DNA fragments of interest do not carry ideal restriction enzyme sites and thus demand end modification. This often introduces small insertions that modify the primary sequence of the final construct (Fig. 2). Furthermore, repeated use of fragments is often hindered by the fact that new inserts contain enzymes that were used before (Fig. 2, we used *Bam*HI and *Eco*RI, which we introduced into protein 2, ORF2). The consequence of this situation is that the fragments prepared cannot be used. In our case, 2 of the previous inserts had to be re-engineered (Fig. 2, *Ptub2*, GFP). Thus, placing the two open reading frames under the α-tubulin promoter and fusing them to GFP required the generation of 8 fragments and the introduction of 10 short sequence stretches.

When cloning the same constructs using YRBC, the vector can be linearized with any single suitable restriction enzyme (Oldenburg et al., 1997). The presence of the 30 bp overlapping sequences, attached by PCR, guarantee directed recombination of all 4 fragments in a single step *in vivo* (Fig. 2). Neither additional sequences are added, nor is the insert DNA altered in any way. This minimizes potential cloning artifacts. In addition, most fragments can be used in both constructs (Fig. 2), which reduces the number of newly-generated inserts to 6. This advantage is even more obvious when more open reading frames are cloned in a similar way. In addition, YRBC is a very cost effective method. In this paper we used “home-made” reagents. For instance, we have replaced the expensive gel purification kit with silica glass suspension, as described previously, with slight modifications (Boyle and Lew, 1995; see materials and methods for details). The yeast plasmid isolation and plasmid miniprep systems were also replaced with home-made solutions, as described elsewhere (Birnboim and Doly, 1979; Gietz and Schiestl, 2007; Hoffman and Winston, 1987; see materials and methods for details). Furthermore, we prepared transformation-competent *E. coli* and *S. cerevisiae* ourselves and transformations were carried out as described previously with slight modifications (Hoffman and Winston, 1987; Tu et al., 2005; see materials and methods for details). In summary, YRBC is a low-cost and efficient way of generating complex constructs, with minimum risk of unwanted sequence modifications. In this way, it is superior to more conventional restriction enzyme-based cloning methods.

### 3.3 Vectors for yeast recombination-based cloning of constructs for use in *Z. tritici*

**Fig. 3:**
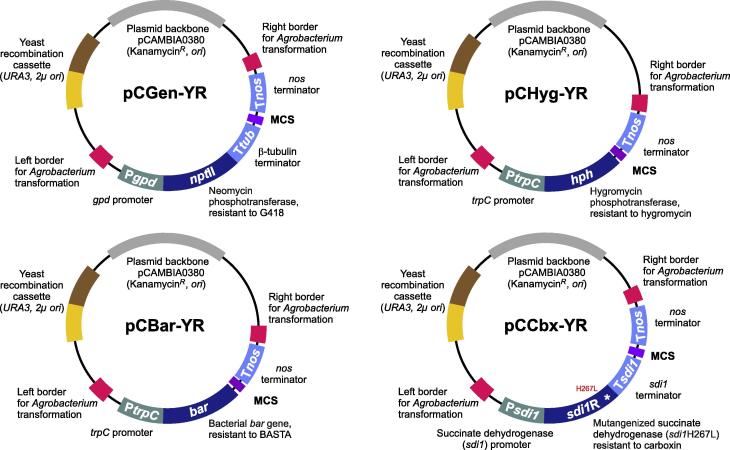
Organization of four cloning vectors for yeast recombination-based cloning in *Z. tritici*. Note that fragments are not drawn to scale. The multiple cloning site is indicated by “MCS”. Vectors need to be linearized by restriction enzyme-based digestion. See main text for further details on fragment sizes and methodology.

*Agrobacterium tumefaciens*-based transformation is well-established for *Z. tritici* (Zwiers and De Waard, 2001). The binary vector pCAMBIA03800 is widely used for this method and it allows replication in *E. coli* and *A. tumefaciens* due to the presence of their corresponding origin of replications (CAMBIA, Canberra, Australia). To make this vector suitable for YRBC, we introduced “yeast survival elements”, – the 2-micron origin of replication (2μ *ori*) and an auxotrophic selectable marker – URA3 (Joska et al., 2014; Kuijpers et al., 2013). Introducing a ∼3.0 kb fragment, consisting of 2μ *ori* and URA3, into plasmids for use in *U*. *maydis* and *M. oryzae* had previously made these vectors suitable for YRBC (Dagdas et al., 2012; Schuster et al., 2011a). We followed a similar strategy here and modified the previously published vectors pCGEN, pCHYG, pCAMB-BAR (Kramer et al., 2009; Motteram et al., 2009, 2011; Perez-Nadales et al., 2014), which carry different dominant selectable markers genes that confer resistance against hygromycin, geneticin and Basta, respectively. The resulting *Z. tritici* vectors pCGEN-YR, pCHYG-YR, pCBAR-YR and pCCBX-YR, are suitable for YRBC and subsequent transformation into *Z. tritici* (Fig. 3).

Furthermore, we generated the vector pCCBX-YR, which contains a mutated allele of the succinate dehydrogenase gene that confers resistance against carboxin (Bowler et al., 2010; Scalliet et al., 2012; see Kilaru et al., 2015). Herein, we provide more detailed description of each vector. Technical details of their generation can be found in the Materials and Methods.

**pCGEN-YR**. The plasmid pCGEN contains the *nptII* gene (795 bp) encoding neomycin phosphotransferase, conferring resistance against Geneticin (Jimenez and Davies, 1980), under the control of *Cochliobolus heterostrophus gpdI* promoter (368 bp) and *Neurospora crassa β-tubulin* terminator (261 bp) sequences (Motteram et al., 2011; Fig. 3). In order to amend the pCGEN for YRBC, a 2820 bp fragment, covering the URA3 marker and 2μ *ori* from plasmid pNEB-hyg-yeast (digested with *Sac*II and *Ssp*I) (Schuster et al., 2012) was ligated *in vitro* with 8257 bp fragment of vector pCGEN (digested with *Sac*II and *Psi*I) resulting in pCGEN-YR. pCGEN vector was built on the *Agrobacterium* binary vector pCAMBIA0380 (CAMBIA, Canberra, Australia). This vector allows *A. tumefaciens*-based transformation into *Z. tritici*, which is based on the 25 bp imperfect directional repeat sequences of the T-DNA borders (right and left border, RB and LB; Fig. 3). The vector also carries a kanamycin resistance gene and origins of replication for amplification in *E. coli* and *A. tumefaciens*.

**pCHYG-YR***.* The plasmid pCHYG contains *hph* gene (1026 bp), encoding hygromycin phosphotransferase, conferring resistance against hygromycin (Waldron et al., 1985), under the control of *Aspergillus nidulans trpC* promoter (361 bp) and *A. tumefaciens nos* terminator (253 bp) sequences (Motteram et al., 2009; Fig. 3). In order to amend the pCHYG for YRBC, a 2820 bp fragment covering the URA3 marker and 2μ *ori* from plasmid pNEB-hyg-yeast (digested with *Sac*II and *Ssp*I) (Schuster et al., 2012) was ligated *in vitro* with 8117 bp fragment of plasmid pCHYG (digested with *Sac*II and *Psi*I) resulting in pCHYG-YR. As this vector was derived from the binary vector pCAMBIA0380 (CAMBIA, Canberra, Australia), it is suitable for *A. tumefaciens*-based transformation into *Z. tritici*.

**pCBAR-YR**. The plasmid pCAMB-BAR contains the *bar* gene (552 bp) encoding phosphinothricin acetlytransferase, conferring resistance against Bialaphos and BASTA (Block et al., 1987), under the control of *A. nidulans trpC* promoter (383 bp) and *A. tumefaciens nos* terminator (253 bp) sequences (Kramer et al., 2009; Fig. 3). In order to amend the pCAMB-BAR for YRBC, a 2847 bp fragment, covering the URA3 marker and 2μ *ori* from plasmid pCGEN-YR (digested with *Bcl*I and *Dra*I) was ligated with 7616 bp fragment of plasmid pCAMB-BAR (digested with *Bcl*I and *Psi*I) resulting in pCBAR-YR. Like the other vectors, pCAMB-BAR is suitable for *A. tumefaciens*-based transformation into *Z. tritici*.

**pCCBX-YR**. The vector pCCBX-YR contains *sdi1^R^* gene (1008 bp) encoding mutant allele of the succinate dehydrogenase, which confers resistance to the fungicide carboxin, under the control of *Z. tritici sdi1* promoter (1027 bp) and the *sdi1* terminator (197 bp) sequences (Fig. 3). This vector was constructed by using homologous recombination in yeast (for details see Methods section). The 9766 bp fragment of pCGEN-YR (digested with *Bam*HI and *Zra*I), 1027 bp of *sdi1* promoter, 1008 bp of H267L mutated allele of *sdi1* gene and 197 bp of *sdi1* terminator were recombined in yeast *S. cerevisiae* to obtain the plasmid pCCBX-YR. 1027 bp *sdi1* promoter and 902 bp 5′ end of the *sdi1* gene was amplified by using primers SK-Sep-282 and SK-Sep11 (Table 1); 106 bp 3′ end of the gene and 197 bp *sdi1* terminator was amplified by using SK-Sep-12 and SK-Sep-283 primers (Table 1). The point mutation (H267L) was introduced with in the 30 bp of regions needed for homologous recombination of both SK-Sep-11 and SK-Sep-12 primers (Table 1). Apart from the *sdi1* promoter and *sdi1* terminator, a point mutation (H267L) was introduced in the *sdi1* gene and the same was also recombined in a single cloning step, demonstrating the powerfulness of the YRBC approach. This vector was also built on the binary vector pCAMBIA0380 (CAMBIA, Canberra, Australia) thus enabling *A. tumefaciens*-based transformation into *Z. tritici.*

## 4 Conclusion

In this study we introduce YRBC as a powerful method of constructing complex vectors for use in *Z. tritici*. This method offers several advantages over conventional restriction enzyme-based cloning: (1) It allows efficient cloning, as many DNA fragments can be used in various combinations (see Fig. 2 for illustration), which also enables the rapid exchange of genes, promoters and dominant selectable marker cassettes; the method (2) is independent of restriction sites, making cloning of large or many fragments easier; (3) allows precise cloning without alteration of the coding sequence; (4) YRBC comes with relatively low associated costs. YRBC was successfully used in studying *U. maydis* (Bielska et al., 2014; Higuchi et al., 2014; Schuster et al., 2011b, 2012; Steinberg et al., 2012). Adapting this powerful cloning method in *Z. tritici* will rapidly inflate the number of useful constructs, required to better understand cell biology and plant invasion strategies in this important wheat pathogen.

## Figures and Tables

**Table 1:** Primers used in this study.

Primer name	Direction	Sequence (5′ to 3′)^a^
SK-41	Sense	GTGGATGATGTGGTCTCTACAGG
SK-Sep-11	Antisense	*ATTCAGAATGGTGAGGCATCGGTACAAGCT*CATGCTGTTGTTGAGTGCGTCC
SK-Sep-12	Sense	*AGCTTGTACCGATGCCTCACCATTCTGAAT*TGCTCAAGGACCTGCCCCAAG
SK-Sep-137	Antisense	CCCGATCTAGTAACATAGATGACA
SK-Sep-282	Sense	*GCTTGACGACATTCCGAAACCCCCAATTTC*GCTACCGAGCGGCGAGCAGA
SK-Sep-283	Antisense	*GCTTGCATGCCTGCAGGTCGACTCTAGAGGATCC*CTTCCGTCGATTTCGAGACAGC

a *Italics* indicate part of the primer that is complementary with another DNA fragment, to be ligated by homologous recombination in *S. cerevisiae.*
